# Zuojin capsule improves T cell exhaustion and tumor immune microenvironment of hepatocellular carcinoma through the mTOR-eIF4E/p70S6K-CDK1 pathway

**DOI:** 10.3389/fimmu.2025.1617604

**Published:** 2025-09-22

**Authors:** Liyuan Hao, Shenghao Li, Jiali Deng, Xiaoyu Hu

**Affiliations:** ^1^ School of Clinical Medicine, Chengdu University of Traditional Chinese Medicine, Chengdu, Sichuan, China; ^2^ Department of Infectious Diseases, Hospital of Chengdu University of Traditional Chinese Medicine, Chengdu, Sichuan, China; ^3^ Department of Integrated Traditional Chinese and Western Medicine Oncology, The Fourth Hospital of Hebei Medical University, Shijiazhuang, Hebei, China

**Keywords:** ZJC, HCC, mTOR, tumor immune microenvironment, TeX

## Abstract

**Background & Aims:**

Hepatocellular carcinoma (HCC) is a major health concern. T cell exhaustion (Tex), a state of T cell dysfunction characterized by reduced effector function and increased expression of inhibitory receptors. This study aimed to explore the mechanism by which Zuojin capsule (ZJC, *Coptidis Rhizoma* and *Evodiae Fructus*) treats HCC and improves Tex.

**Methods:**

To identify HCC-related and Tex-associated targets, two HCC expression microarray datasets were integrated. Targets related to Tex were retrieved from GeneCards and Online Mendelian Inheritance in Man (OMIM) databases. Active compounds in ZJC were screened. A protein-protein interaction (PPI) network of overlapping targets was constructed using STRING to identify core functional modules. To verify the anti-proliferative effect of ZJC on HCC cells, the CCK-8 assay was performed to detect the viability of Hep3B and HepG2.2.15 cells treated with gradient concentrations of ZJC. Western Blot analysis was conducted to measure the protein expression levels of key molecules. Immunohistochemical (IHC) staining was used to assess the proliferation index of tumor cells, the infiltration of immune cells, and the expression of immune-related markers.

**Results:**

HCC-related genes, Tex targets, and ZJC targets were identified through bioinformatics analysis, 136 overlapping targets were obtained. ZJC inhibited Hep3B/HepG2.2.15 cell proliferation with IC_50_ values of 310 μg/mL and 530 μg/mL, respectively. The pathway analysis conducted using DAVID revealed that the intersecting targets were mainly enriched in the mTOR signaling pathway and the transcriptional regulation process. H22 xenografts were treated with ZJC or anti-PD-1 to evaluate tumor growth and immune responses. ZJC suppressed HCC cell proliferation and reduced the expression of Ki67. Mechanistically, it downregulated phosphorylated mTOR (p-mTOR), p-eIF4E, and p-p70S6K, and this downregulated state could be reversed by the restoration of mTOR activators. ZJC reduced the expression of cyclin-dependent kinase 1 (CDK1). In HCC tissues, M1 macrophages were reduced, while M2 macrophages and exhausted T cells were accumulated. ZJC treatment inhibited tumor growth and modulated immune infiltration. Additionally, ZJC and anti-PD-1 promoted the expression or aggregation of CD8-positive cells. In addition, the control group showed relatively high positive staining for CD163, whereas ZJC and anti-PD-1 inhibited the expression or aggregation of CD163-positive cells.

**Conclusion:**

ZJC exerts dual anti-tumor effects by inhibiting the mTOR-eIF4E/p70S6K-CDK1 pathway and remodeling the immunosuppressive microenvironment of HCC.

## Introduction

Hepatocellular carcinoma (HCC), the most common primary malignant tumor of the liver, imposes an enormous disease burden globally. Primary liver cancer ranks as the six most common cancer globally and is the third leading cause of cancer-related mortality (1). HCC is the most prevalent form of primary liver cancer, accounting for approximately 90% of cases. Despite certain progress in the treatment of HCC in modern medicine, such as surgical resection, local ablation, transarterial chemoembolization (TACE), and the application of various targeted and immunotherapeutic drugs, the overall prognosis of patients remains unsatisfactory (2). A large proportion of HCC patients are diagnosed at intermediate to advanced stages, thereby losing eligibility for curative surgical interventions. Moreover, the recurrence rate is high, and the 5-year survival rate is less than 20% (3). Therefore, a deeper understanding of the pathogenesis of HCC and the development of more effective therapeutic strategies have become critical priorities in HCC.

The tumor microenvironment (TME) supports HCC initiation and progression via interactions between tumor cells, immune cells, stromal cells, and extracellular matrix (4, 5). T cell exhaustion (Tex) is a key immunosuppressive phenomenon in the TME, characterized by the progressive impairment of T-cell function. This is manifested by diminished proliferative capacity, reduced cytokine secretion, and impaired cytotoxic activity against tumor cells (6). It is linked to factors like continuous antigen stimulation and upregulated inhibitory immune checkpoint molecules in the TME (7, 8). Targeting Tex is a critical frontier in immunotherapy.

Traditional Chinese medicine has a long-standing history and a unique theoretical system in disease treatment, providing abundant drug resources and treatment concepts for modern medicine. Zuojin capsule (ZJC), a classic Chinese herbal compound, is composed of two herbs, namely *Coptidis Rhizoma* (Huang-lian in Chinese) and *Evodiae Fructus* (Wu-zhu-yu in Chinese) (9). The ratio of the two is 6:1. *Coptidis Rhizoma* is derived from the dried rhizome of *Coptis chinensis* Franch and exhibits multiple pharmacological effects, such as anti-inflammatory and anti-cancer properties (10). *Evodiae Fructus* is obtained from the immature fruit of *Evodia rutaecarpa* Benth (11) and is widely used in the treatment of inflammation and cancer (12). In traditional medicine, ZJC is commonly used for “liver fire invading the stomach” — a condition where poor liver function causes stagnation of its vital energy (“qi”), which turns into pathological “fire” and disrupts stomach digestion, leading to symptoms like burning stomach pain and irritability (13). In recent years, an increasing number of studies have indicated that ZJC shows potential efficacy in the treatment of various diseases, including gastrointestinal disorders and tumors (14, 15). Notably, ZJP significantly inhibits tumor growth in an orthotopic HepG2 xenograft mouse model with an intact immune function (16). However, the mechanism of action of ZJC in the treatment of HCC remains incompletely understood.

Network pharmacology, an emerging interdisciplinary field integrating systems biology, bioinformatics, and other techniques, analyzes drug mechanisms by constructing “drug-target-disease” networks, supporting traditional Chinese medicine research (17, 18). Its predictions can be further validated through *in vitro* and *in vivo* experiments.

This study combines network pharmacology with experimental techniques to explore the mechanism of ZJC in treating HCC, particularly its association with Tex. By analyzing gene chip data, screening database targets, constructing PPI networks, and conducting bioinformatics analysis, key targets and pathways are identified. The effects and mechanisms of ZJC on HCC cells and tumor growth are then verified, aiming to provide new insights for clinical HCC treatment and promote the application of traditional Chinese medicine in oncology.

## Materials and methods

### Collection of liver cancer microarray data

The original microarray datasets GSE45436 (19) and GSE121248 (20) of HCC were downloaded from the Gene Expression Omnibus (GEO) (https://www.ncbi.nlm.nih.gov/geo/) database (21). The sequencing platform for both microarrays were “GPL570” [HG-U133_Plus_2] Affymetrix Human Genome U133 Plus 2.0 Array. The sequencing data from two microarrays of HCC tissues on the same sequencing platform were analyzed and processed using “GEOquery” and “tidyverse” package, and then merged after normalization. The ComBat function of the “sva” package was employed to eliminate batch effects (22). Principal component analysis (PCA) was used to compare data characteristics before and after normalization. While the raw dataset showed scattered distribution and ambiguous clustering, the normalized dataset exhibited significantly improved distribution and more distinct clustering patterns (**Supplementary Figure 1**).

### Differentially expressed genes analysis

The expression profile data of the gene microarray were analyzed using the “limma” package in R Studio software to screen out the differentially expressed genes (DEGs). The screening criteria for DEGs were *P* < 0.05 and |log2 (fold-change) | > 1. Subsequently, the volcano plot and heatmap were drawn by using the “ggpubr” package and “Pheatmap” package of R, respectively.

### Acquisition of T cell exhaustion targets

The targets related to Tex were obtained from the GeneCards (https://www.genecards.org/) database and Online Mendelian Inheritance in Man (OMIM) (https://omim.org/) database (23). All databases used the keywords “T cell exhaustion”. Venny 2.1.0 (https://bioinfogp.cnb.csic.es/tools/venny/) (24) online tool was used to draw Venn diagram to realize the core target between ZJC, HCC and Tex.

### Acquisition of ZJC targets

By searching the Traditional Chinese Medicine Systems Pharmacology Database and Analysis Platform (25) (TCMSP, https://old.tcmsp-e.com/tcmsp.php) database, the ZJC chemical components related to related to the two Chinese herbs, namely *Coptidis Rhizoma* and *Evodiae Fructus* were retrieved. The retrieval results were screened, with parameters being the comprehensive oral bioavailability (OB) and drug-likeness (DL). OB represents the percentage of the drug reaching the systemic circulation at the same oral dose. DL is used to evaluate the “drug-likeness” degree of the required compound, which helps optimize the pharmacokinetics and drug properties of the drug, such as solubility and chemical stability (26). Subsequently, the chemical components of traditional Chinese medicine that simultaneously met the criteria of OB>30% and DL>0.18 (27, 28) were collected. Then the targets that met the criteria were selected. Moreover, these data were combined and duplicate items were deleted. Finally, the targets of traditional Chinese medicine collected from the database were obtained and regarded as the potential targets of ZJC.

### Construction of PPI network

The intersection targets of ZJC, HCC, and Tex were imported into the STRING (29) (https://cn.string-db.org/) database for PPI analysis, and a PPI network diagram was constructed. Subsequently, the PPI network diagram was imported into the Cytoscape software for visual analysis.

### Immune infiltration analysis

To further evaluate the immune cell infiltration in normal tissues and liver cancer tissues, we utilized xCell (30) (https://xcell.ucsf.edu/) to assess the composition of various immune cells in normal tissues and liver cancer tissues. The results of the correlation between gene expression and immune cells were presented as box plots. The xCell database integrates the advantages of gene enrichment analysis through back-folding and can evaluate 64 cell types, covering a wide range of adaptive and innate immune cells, hematopoietic progenitor cells, epithelial cells, extracellular stromal cells, and cells related to the TME. Subsequently, Spearman correlation analysis was further employed to analyze the correlation between 5 key genes and some key immune cells, and the results were displayed as lollipop plots.

### Functional and pathway analysis

To elucidate the functional roles and pathway involvement of the intersecting targets, Gene Ontology (GO) and Kyoto Encyclopedia of Genes and Genomes (KEGG) analyses were performed using the R package “clusterProfiler” and The Database for Annotation, Visualization and Integrated Discovery (DAVID) (31) (https://david.ncifcrf.gov/). GO analysis encompassed three categories: biological process (BP), cellular component (CC), and molecular function (MF) (32). Terms with *P*<0.05 were considered statistically significant.

### Identification of survival-associated genes

To identify survival-associated genes in HCC, RNA-seq expression data from tumor and normal tissues were retrieved from The Cancer Genome Atlas (TCGA) and Genotype-Tissue Expression (GTEx) databases using GEPIA 2 (http://gepia2.cancer-pku.cn/#index). Survival analysis was performed on HCC-related genes to evaluate their prognostic significance. Genes with statistically significant associations with survival outcomes were identified as candidate genes for further study.

### Expression, staging, and prognostic significance of five key targets

Gene expression and staging analyses in normal liver tissues and HCC tissues were conducted using GEPIA 2 (33) and the University of ALabama at Birmingham CANcer data analysis Portal (UALCAN, http://ualcan.path.uab.edu/analysis.html) (34). The Kaplan-Meier Plotter database (35) (https://kmplot.com/analysis/) provides survival-related information for more than 54,000 genes in 21 types of cancer. It was used to analyze the relationship between five key genes and the overall survival (OS) of HCC patients. The screening criteria included hazard ratio (HR) within the 95% confidence interval (CI) and log-rank *P* value <0.05.

### Correlation analysis

The GEPIA 2 (http://gepia2.cancer-pku.cn/#correlation) database integrates a large amount of gene expression data from tumors and normal tissues. In this study, gene correlation analysis was conducted using the GEPIA 2 database. During the research process, we conducted correlation analyses between cyclin-dependent kinase 1 (CDK1) and programmed death receptor-1 (PD-1), CDK1 and programmed death receptor ligand-1 (PD-L1), CDK1 and cytotoxic T lymphocyte-associated antigen-4 (CTLA-4), CDK1 and T-cell Ig and ITIM domain (TIGIT), CDK1 and T-cell immunoglobulin and mucin-domain containing-3(TIM-3), as well as CDK1 and lymphocyte-activation gene 3 (LAG-3). The relationship was determined based on the correlation coefficients and *P* values provided by the database.

### Molecular docking

To further verify the effect of ZJC on CDK1, we conducted a molecular docking verification between the main components of ZJC and CDK1. The 3D structure of CDK1 and the main components structure of ZJC were obtained from the RCSB PDB (http://www.rcsb.org/) and PubChem (https://pubchem.ncbi.nlm.nih.gov/) databases. Water molecules and the original ligands were removed by using the PyMOL software. Then, the target protein was imported into the AutoDock Tools 1.5.6 software for hydrogenation treatment. The results were stored in the PDBQT format. AutoDock Vina was run for molecular docking, and the results were visualized using PyMOL.

### Reagents

ZJC was purchased from Haihe Pharmaceutical Co, LTD (production batch number: 20191004, specification: 350mg/seeds) (Wenzhou, China) (9). Anti-PD-1 (BE0146) and anti-PD-1 (66220-1-Ig) were purchased from Bioxcell and Proteintech Group, Inc, respectively. Anti-Ki67 (HA721115) and anti-p-p70S6K (HA721803) were purchased from HUABIO. Anti-CD8 (29896-1-AP) and anti-CD163 (83285-4-RR) were purchased from Proteintech. Anti-β-actin (AC026), anti-mTOR (A2445), and anti-p-mTOR (AP0115) were purchased from Abclonal. Anti-CDK1 (CY5061), anti-eIF4E (CY8863), anti-p-eIF4E (CY5419), and anti-p70S6K (CY5365) were purchased from Abways. Goat Anti-Rabbit IgG (H+L) HRP (S0001) was purchased from Affinity. The BCA kit (P0011) was purchased from Beyotime. The ECL chemiluminescence substrate (BL520B) was purchased from Biosharp.

### Cell line and treatment

Hep3B2.1-7 (Hep3B) and HepG2.2.15 cells were purchased from icell and cultured in cell complete culture medium (icell-h092-001b, icell) supplemented with 10% fetal bovine serum (FBS, C04001-500, Vivacell), 100 U/ml penicillin and 100 μg/ml streptomycin at 37°C and 5% CO_2_ in an atmosphere of 100% humidity.

### Cell viability assay

Hep3B and HepG2.2.15 cells were seeded in 96-well plates (1×10^4^ cells/well). They were treated with ZJC (25, 50, 100, 150, 200, 300, 400μg/mL), at different concentration for 24 hours, and 48 hours, respectively. Cell viability was determined with Cell Counting Kit-8 (CCK-8, BS350B, Biosharp) according to the manufacture’s protocol. Finally, optical density was monitored IC50 values were obtained from the cytotoxicity curves using the ELx800 software at 450 nm. IC50 values were obtained from the cytotoxicity curves using the SOFTmax PRO software.

### Establishment of syngeneic tumor models

To further evaluate the anti-tumor efficacy of ZJC *in vivo*, a syngeneic tumor model was established in male C57BL/6 mice (4–5 weeks old, 18–20 g) purchased from Jiangsu Ailingfei Biotechnology Co., Ltd. (License No. SCXK (Su) 2023-0020). The experimental protocol was approved by the Institutional Animal Care and Use Committee of the Affiliated Hospital of Chengdu University of Traditional Chinese Medicine (No. 2021DL-019). All mice were housed in a specific pathogen-free (SPF) environment under controlled conditions (temperature: 22 °C, humidity: 65%, 12-hour light/dark cycle) with free access to food and water. After a 7-days acclimatization period, the mice were fasted for 12 hours (water permitted) prior to the experiment. This fasting step was intended to minimize the interference of food residues in the abdominal cavity on the accuracy of injection site localization during subsequent subcutaneous tumor cell injection, while ensuring that all experimental animals were in a consistent baseline state for the experiment. A total of 1×10^7^ H22 cells were subcutaneously injected into the right flank of the mice to induce tumor formation. The mice were randomly divided into four groups with five mice in each group. The control group was given normal saline by gavage. According to the rules for converting equivalent doses between mice and humans, the dosage per kilogram of body weight for mice is calculated based on 9.01 times the dosage for adults (60 kg) (36). In the low-dose ZJC group (ZJC-L), 0.364 g/kg/d of ZJC was administered by gavage. The high-dose ZJC group (ZJC-H) was given ZJC by gavage at 0.728 g/kg/d. The anti-PD-1 group was intraperitoneally injected with anti-PD-1 monoclonal antibody (10 mg/kg, dissolved in normal saline) every 3 days. Body weight and tumor volume were measured every three days. Tumor volume was calculated by the formula: V (mm^3^) = width^2^ (mm^2^)×length (mm)×0.5 (37). During the treatment, no mice died from loading tumor. For body weight and tumor volume measurements, the researcher conducting the measurements was blinded to group allocation. After 21 consecutive days of treatment, the mice were euthanized, and tumors were excised, photographed, and weighed. All data were recorded for subsequent analysis.

### Hematoxylin and eosin staining

All the organs were promptly removed and then washed with cold phosphate-buffered saline (PBS). The liver tissues were fixed in 4% formaldehyde for 24 hours prior to being embedded in paraffin. The thickness of the sections was 5 µm. The sliced sections were stained with hematoxylin-eosin (H&E), and the changes in histopathology were observed under a microscope (Pannoramic SCAN II).

### Immunohistochemistry

Paraffin-embedded tissue samples were cut into sections with a thickness of 5 micrometers. Immunohistochemical staining was then carried out for anti-Ki67, anti-CD8, anti-CD163, anti-p-eIF4E, anti-p-p70S6K. The sections were developed using a 3,3-diaminobenzidine kit and subsequently stained with hematoxylin. After the staining process, the sections were differentiated. They were then rinsed with an antiblue solution, followed by dehydration and clarification steps. Finally, the sections were sealed. Ultimately, the sections were examined under a microscope at magnifications of 20× and 40× to observe the presence of brown peroxidase in the liver tissue. The immunohistochemistry (IHC) results were evaluated by two senior histopathologists. Cells with their cell membranes or cytoplasm stained in light yellow or tan colors were regarded as positive cells. The optical density of the stained cells was measured using the Image-Pro Plus 6.0 software, which was manufactured by Media Cybernetics in the United States. The results of immunohistochemistry were examined by 2 senior histopathologists using the double blind method.

### Western blot

Hep3B and HepG2.2.15 cells were seeded in 6-well plates at a density of 1×10^6^ cells per well and then treated as described previously. Briefly, after being washed with PBS, the cells were directly lysed using the lysate. The primary antibodies used included anti-β-actin, anti-CDK1, anti-eIF4E, anti-p-eIF4E, anti-p70S6K, anti-p-p70S6K, anti-mTOR, and anti-p-mTOR. The secondary antibodies were Goat Anti-Rabbit IgG (H+L) HRP. The protein concentration was quantified by using the BCA kit. These protein bands were detected by an enhanced chemiluminescence (ECL) detection system (Bio rad, ChemiDoc XRS^+^). The results were measured by the Image-Pro Plus 6.0 software (from Media Cybernetics).

### Statistical analysis

All data were analyzed and collated using R (version 4.2.1) software and GraphPad Prism 8 software. A *P*-value less than 0.05 was considered to indicate a statistically significant difference. Each experiment was performed in triplicate, with a minimum of three replicates per sample. Data are presented as means ± SD. For comparisons involving more than two groups, one-way ANOVA was applied, followed by *post-hoc* pairwise comparisons. *P*<0.05 was considered statistically significant.

## Results

### Identification of HCC targets

**Figure 1:**
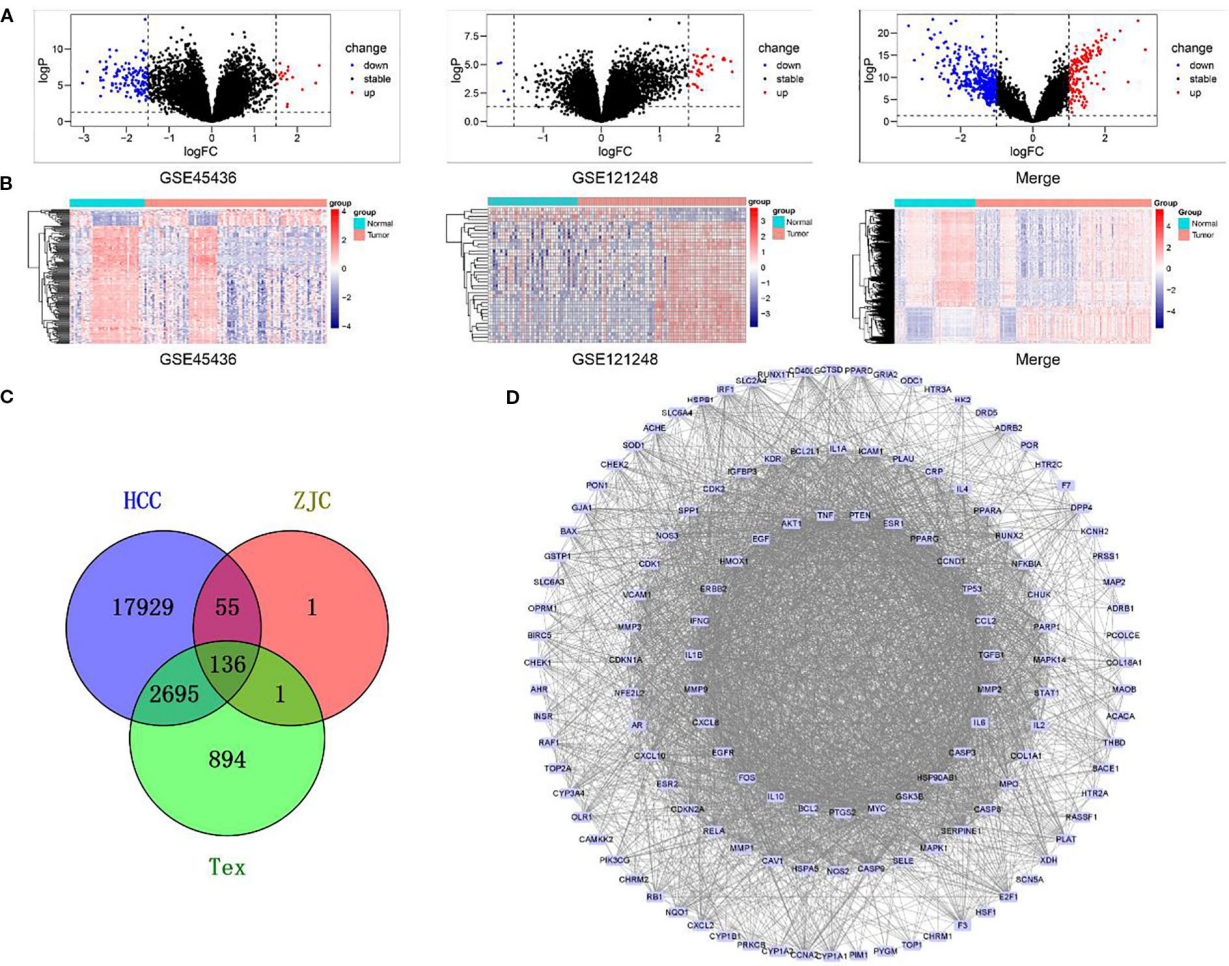
Gene expression analysis and interaction network in different biological groups. **(A)** Volcano plots display the changes in gene expression in different datasets (GSE45436, GSE121248, and the merged dataset). Black dots represent genes with stable expression, red dots represent up-regulated genes, and blue dots represent down-regulated genes. **(B)** Heatmaps show the patterns of gene expression in GSE45436, GSE121248, and the merged dataset. **(C)** A Venn diagram illustrates the overlap of genes among the HCC, ZJC, and Tex groups. The numbers represent the unique genes in each group and the genes in the overlapping regions. **(D)** The PPI interaction network depicts the interaction relationships within a specific gene set. Nodes represent genes, and edges represent the interactions between genes.

To identify HCC-related targets, we integrated two HCC expression microarray datasets, GSE45436 and GSE121248. GSE45436 contains 39 normal liver tissues and 95 HCC tissues, and GSE121248 contains 37 normal liver tissues and 70 HCC tissues. After batch effect correction and normalization using the R package “limma”, the merged dataset included 76 normal liver tissues and 165 HCC tissues. Differential expression analysis identified 20,815 significantly dysregulated HCC targets. Volcano plots showed up-regulated and down-regulated genes in HCC and normal tissues, while heat plots showed different expression patterns in the combined cohort (**Figures 1A, B**).

### Acquisition of Tex-related targets

Genes related to Tex were retrieved from the GeneCards database and OMIM database, with 3,330 and 436 targets, respectively. After merging and removing duplicates, 3,726 targets related to Tex were retained (**Figure 1C**).

### Target identification of ZJC and protein-protein interaction network construction

The effective components in ZJC (*Coptidis Rhizoma* and *Evodiae Fructus*) were screened by TCMSP. According to the screening criteria of OB> 30% and DL> 0.18, 14 compounds (11 with targets) were identified from *Coptidis Rhizoma* and 30 compounds (24 with targets) were identified from *Evodiae Fructus*. The corresponding targets were input into the UniProt database for standardization, resulting in 165 targets for *Coptidis Rhizoma* and 191 for *Evodiae Fructus*. After merging and deleting duplicate data, 193 unique targets were retained as potential therapeutic targets for ZJC. The ZJC target, HCC-related target and Tex-related target were analyzed using Venny 2.1.0, and 136 intersection targets were obtained (Figure1C). These intersection targets showed that ZJC might be a candidate mechanism for exerting anti-HCC effects by regulating Tex. A PPI network consisting of 136 targets and 2910 edges was constructed using the STRING database. This network was visualized in Cytoscape software and key genes were determined by analyzing the degree values (**Figure 1D**).

### ZJC inhibits cell proliferation in Hep3B and HepG2.2.15 cells

**Figure 2:**
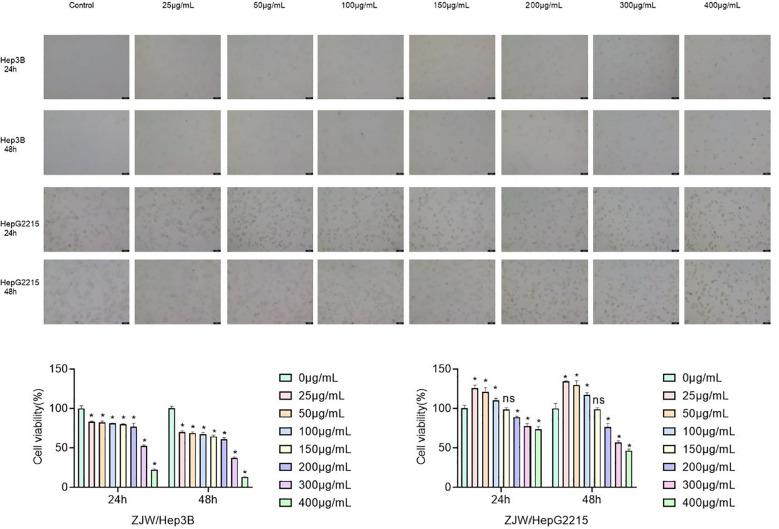
Effect of ZJC at different concentrations on the viability of Hep3B cells and HepG2.2.15 cells. **(A)** The decreased cell number was detected at 24 hours and 48 hours treatment with different concentrations of ZJC. **(B)** CCK8 was used to test the inhibitory effect of ZJC on Hep3B and HepG2.2.15 cell proliferation.

The antiproliferative effect of ZJC on liver cancer lines Hep3B and HepG2.2.15 was evaluated by CCK-8. In Hep3B cells, ZJC treatment significantly inhibited cell proliferation in a dose- and time-dependent manner. At 24 hours, compared with the control group, ZJC at concentrations of 25, 50, 100, 150, 200, 300 and 400μg/mL reduced cell viability. At all test concentrations, this inhibitory effect was further enhanced within 48 hours. In HepG2.2.15 cells, high concentrations (200,300 and 400μg/mL) of ZJC showed significant inhibitory effects at both 24 and 48 hours (**Figure 2A**). The half-maximal inhibitory concentration (IC50) of Hep3B and HepG2.2.15 cells treated with ZJC for 24 hours was approximately 310μg/mL and 530μg/mL, respectively (**Figure 2B**).

### ZJC inhibits HCC by mTOR signaling pathway

**Figure 3:**
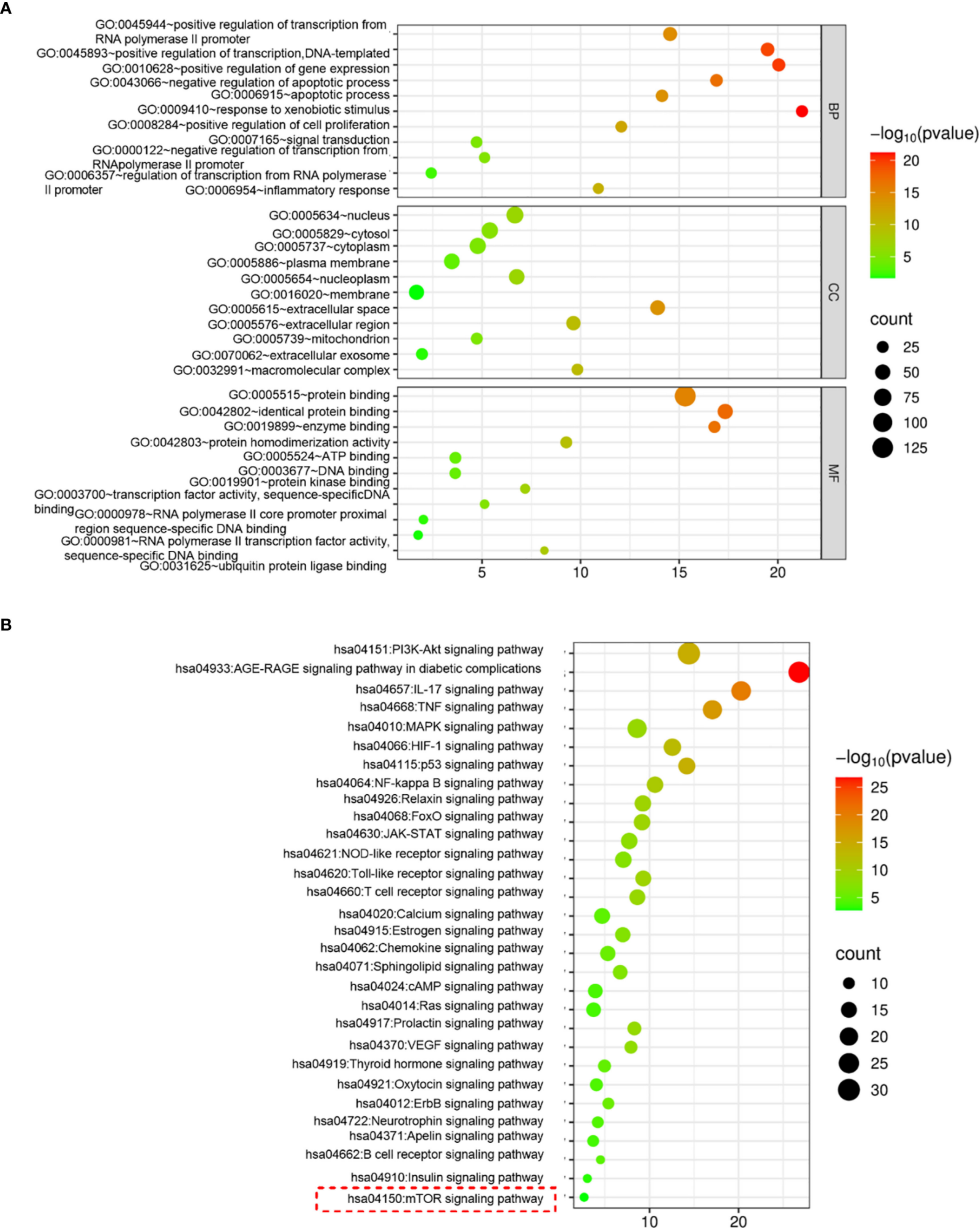
ZJC inhibits HCC through the mTOR signaling pathway. **(A)** GO functional enrichment analysis. **(B)** KEGG pathway enrichment analysis.

To further clarify the mechanism by which ZJC inhibits HCC, the intersection targets were further imported into the DAVID database for GO and KEGG analyses. Only the top 33 results are presented in the **Figure 3**. The results in the BP of GO indicated that the intersection targets were mainly enriched in GO:0045944~positive regulation of transcription from RNA polymerase II promoter, GO:0045893~positive regulation of transcription, DNA-templated, etc. In the CC of GO, the intersection targets were mainly enriched in GO:0005634~nucleus, GO:0005829~cytosol, etc. In the MF of GO, the intersection targets were mainly enriched in GO:0005515~protein binding, GO:0042802~identical protein binding, etc. (**Figure 3A**). KEGG pathway analysis showed that the intersection targets were mainly enriched in hsa04150: mTOR signaling pathway, hsa04621: NOD-like receptor signaling pathway, hsa04151: PI3K-Akt signaling pathway, etc. (**Figure 3B**).

**Figure 4:**
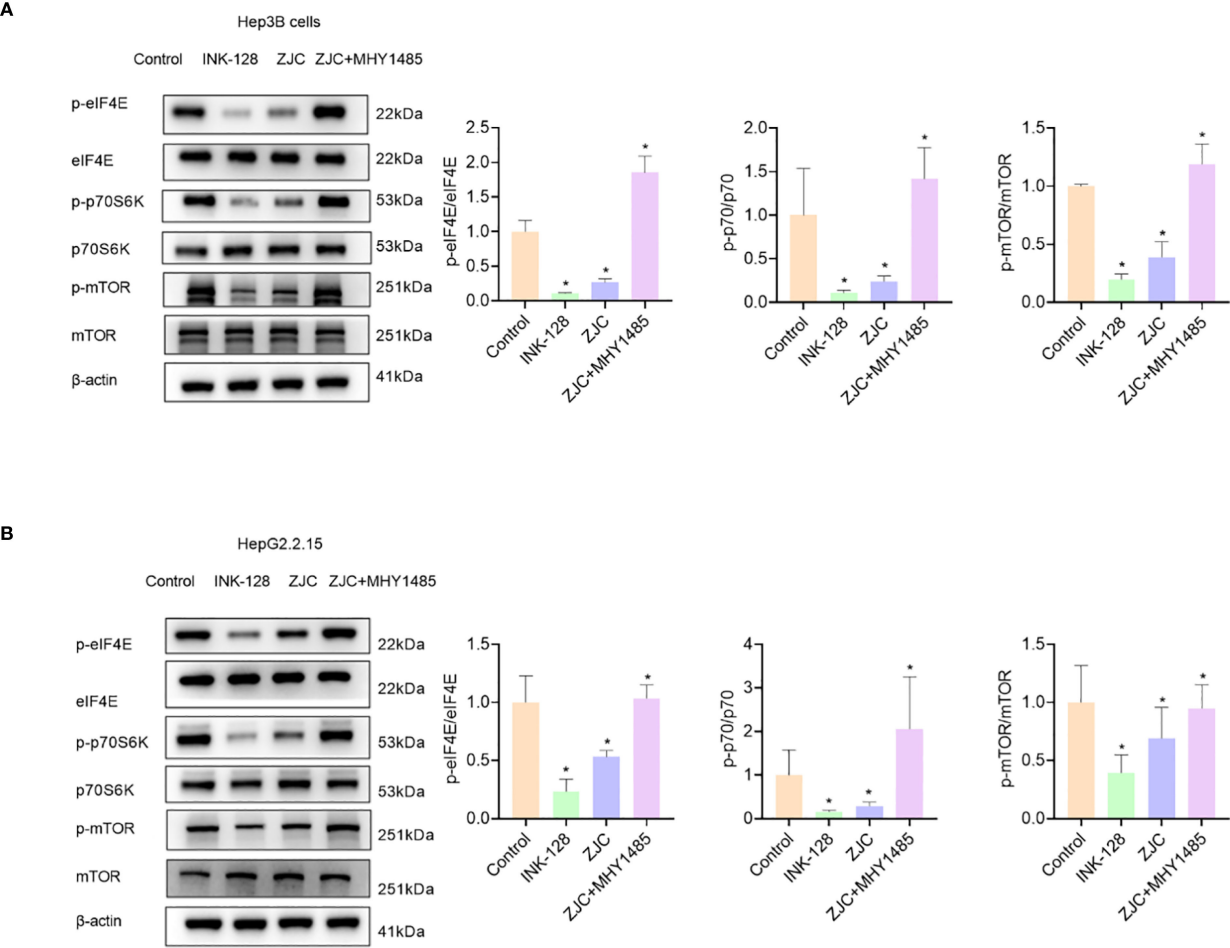
ZJC inhibits HCC by suppressing the mTOR signaling pathway. **(A)** The expression of p-eIF4E, eIF4E, p-p70S6K, p70S6K, p-mTOR and mTOR proteins were detected by Western blot assay in Hep3B cells. **(B)** The expression of p-eIF4E, eIF4E, p-p70S6K, p70S6K, p-mTOR and mTOR proteins were detected by Western blot assay in HepG2.2.15 cells.

To further identify the key pathways of ZJC in the treatment of HCC, *in vitro* experiments were carried out for verification. The protein results showed that in Hep3B cells, compared with the control group, the mTOR inhibitor (INK128) and ZJC decreased the expression of p-mTOR, as well as the expression of two key molecules downstream of the mTOR pathway, p-eIF4E and p-p70S6K. When ZJC was used in combination with the mTOR activator (MHY1485), this inhibitory effect was restored (38–40) (**Figure 4A**). Similarly, in HG2215 cells, compared with the control group, INK128 and ZJC decreased the expression of p-mTOR, as well as the expression of p-eIF4E and p-p70S6K. When ZJC was combined with MHY1485, this inhibitory effect was restored (**Figure 4B**).

### CDK1 is a key target of ZJC in inhibiting HCC

**Figure 5:**
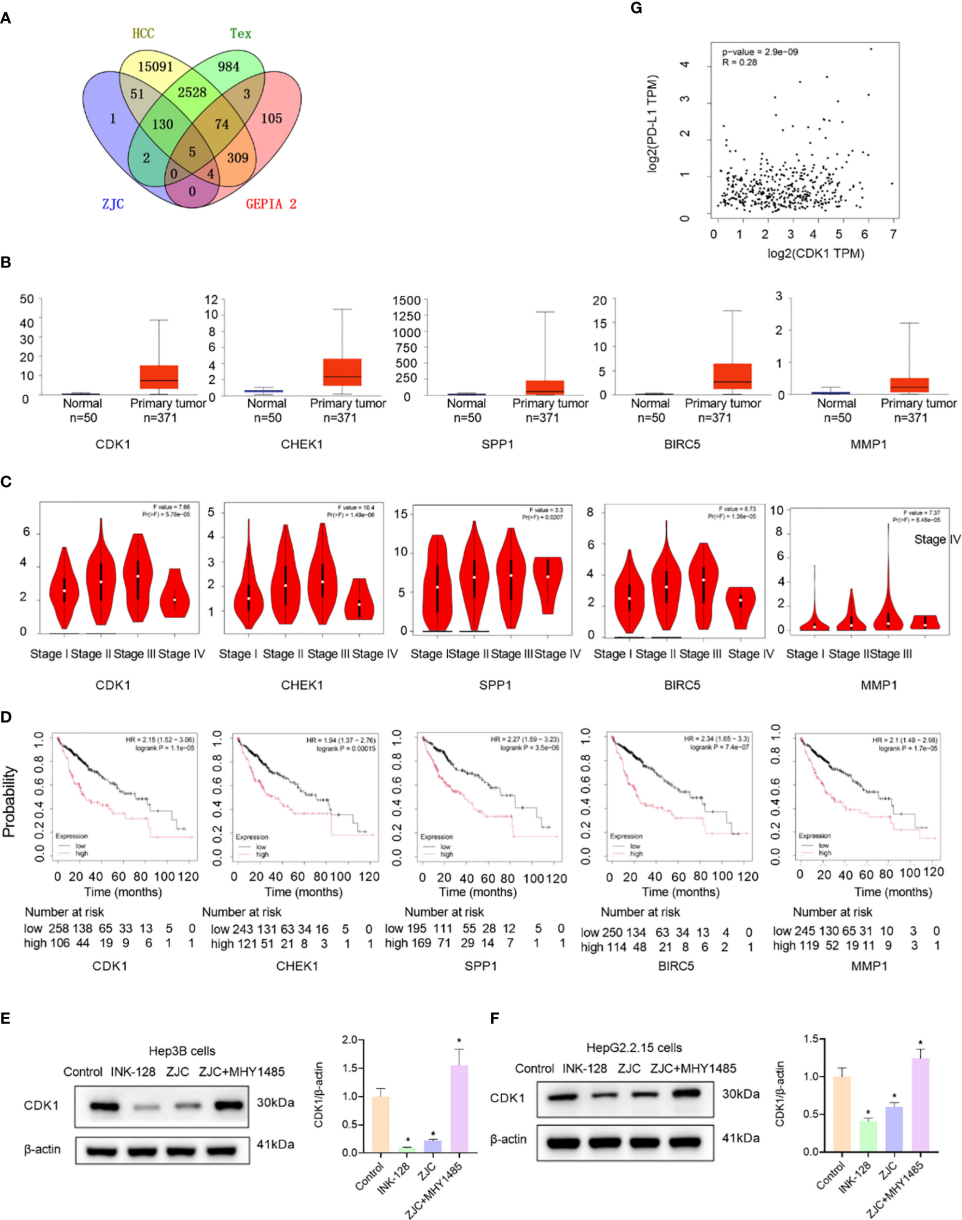
Identification and validation of key genes. **(A)** Venn diagram shows the overlap of genes among ZJC, Tex, HCC, and GEPIA 2 datasets. **(B)** Box-plots show the expression levels of CDK1, CHEK1, SPP1, BIRC5, and MMP1 in normal tissues and primary tumor tissues. **(C)** Violin plots depict the distribution of CDK1, CHEK1, SPP1, BIRC5, and MMP1 expression across different stages (I-IV) of HCC. The plots provide insights into the relationship between gene expression and tumor staging. **(D)** Kaplan-Meier survival curves of HCC patients based on the low and high expression levels of CDK1, CHEK1, SPP1, BIRC5, and MMP1. **(E)** The expression of CDK1 proteins were detected by Western blot assay in Hep3B cells. **(F)** The expression of CDK1 proteins were detected by Western blot assay in HepG2.2.15 cells. **(G)** The correlation between CDK1 and PD-L1.

Subsequently, to further identify the key targets of ZJC in the treatment of HCC, we obtained 500 candidate targets using the GEPIA 2 database, and these targets were different survival-related genes. By taking the intersection, 5 survival-related key targets were obtained, including CDK1, checkpoint kinase 1 (CHEK1), secreted phosphoprotein 1 (SPP1), baculoviral inhibitor of apoptosis repeat containing 5 (BIRC5), and matrix metalloproteinase-1 (MMP1) (**Figure 5A**). We further explored the expression of these genes. The results from the UALCAN database showed that the expression of the 5 key targets was upregulated in cancer tissues (**Figure 5B**). The results also indicated that in the GEPIA 2 database, except for stage IV, the expression of these 5 key genes increased with the increase of the stage (**Figure 5C**). Kaplan-Meier Plotter survival analysis demonstrated that high expression of these 5 key genes was associated with poor OS (**Figure 5D**). Studies have shown that mTOR activation promotes the upregulation of CDK1 (41), and rapamycin downregulates the expression of CDK1 by inhibiting the mTOR/p70S6K pathway (42). The results of *in vitro* experiments showed that compared with the control group, INK128 and ZJC decreased the expression of CDK1. The expression of CDK1 was restored after the combined use of ZJC and MHY1485 (**Figures 5E, F**). The GEPIA 2 database showed that the expressions of CDK1 and PD-L1 were positively correlated (*P* = 2.9E-09, r = 0.28) (**Figure 5G**). Through the TCMSP database, we obtained multiple components of ZJC. Among them, the top 4 components in terms of degree values were quercetin, beta-sitosterol, berberine and isorhamnetin. It is generally believed that a docking score of < 0 kcal/mol indicates that the component and the target can spontaneously bind, a score of -4.25 kcal/mol represents good docking affinity, and a score of -7 kcal/mol is considered to have a very strong docking affinity (43). The molecular docking results show that quercetin, beta-sitosterol, berberine and isorhamnetin have good interactions with CDK1. The detailed information of molecular docking is shown in **Supplementary Table 1**. Quercetin forms three hydrogen bonds with CDK1 at the amino acid residues ASP-86, LEU-83. Isorhamnetin forms ten hydrogen bonds with CDK1 at the amino acid residue SER-166, ARG-23, SER-182, GLY-154, LEU-125, ARG-151 (**Supplementary Figure 2**).

### ZJC inhibits growth of tumor *in vivo*

**Figure 6:**
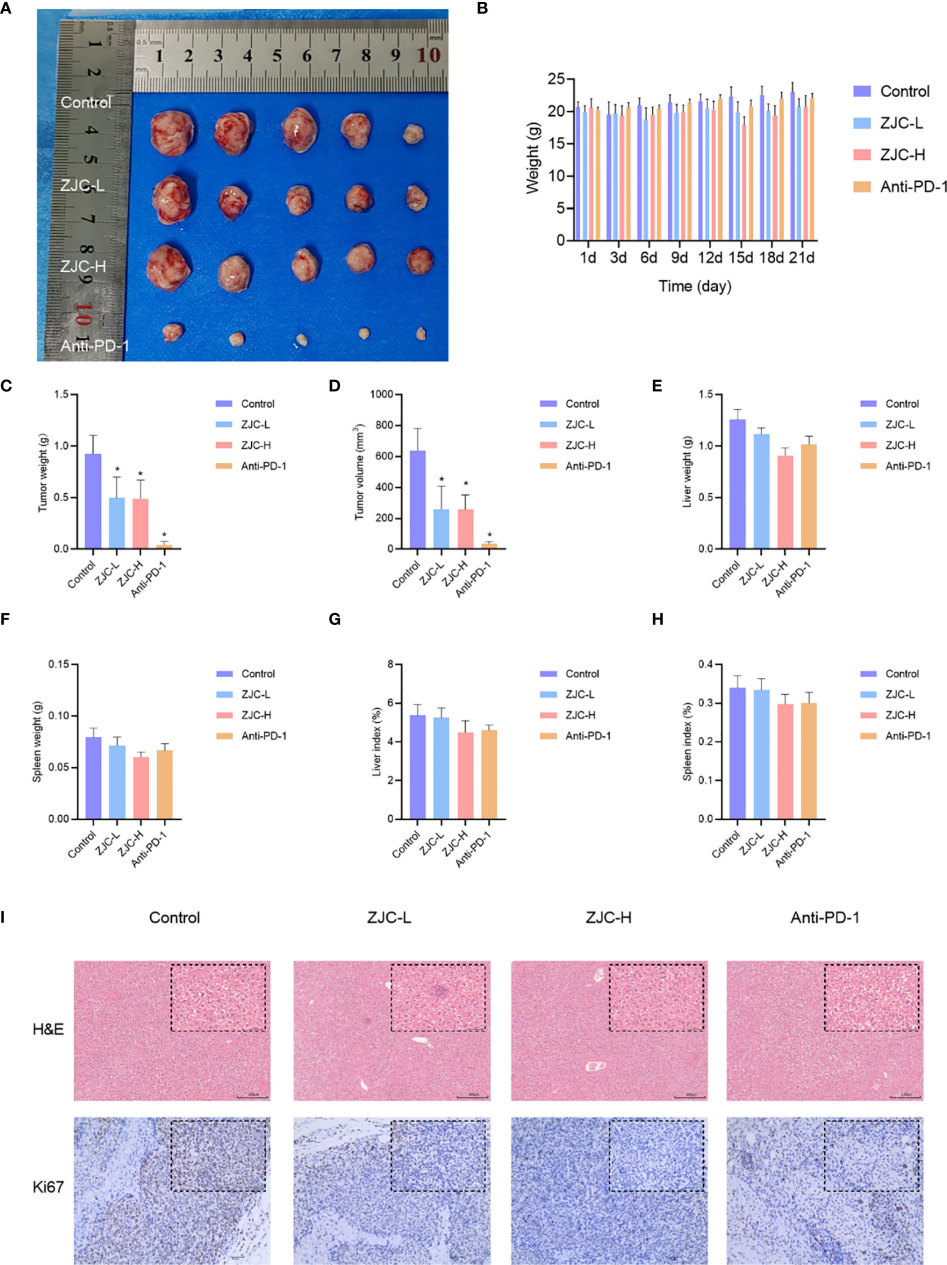
ZJC inhibits the growth of HCC in vivo **(A)** Macroscopic images of tumors from different treatment groups (control, ZJC-L, ZJC-H, and anti-PD-1) in a mouse HCC model. **(B)** The body weight changes of the mice in each group on days 1, 3, 6, 9, 12, 15, 18, and 21. **(C-H)** The bar chart shows the comparison between the control group and the treatment group in terms of tumor weight **(C)**, tumor volume **(D)**, liver weight **(E)**, spleen weight **(F)**, liver index **(G)** and spleen index **(H, I)** H&E and Ki67 staining of each group.

To further evaluate the anti-tumor effect of ZJC *in vivo*, we established a mouse syngeneic tumor model using H22 cells. Compared with the control group, the ZJC-L, ZJC-H, and anti-PD-1 groups inhibited tumor weight and volume (**Figures 6A, C, D**). This result demonstrated that both ZJC treatment and anti-PD-1 treatment exerted an inhibitory effect on tumor growth. There was no significant difference in the final body weight of the mice at the end of the experiment (**Figure 6B**). In addition, there were no significant changes in liver weight, spleen weight, liver index, and spleen index among the four groups of mice (**Figures 6E-H**). In summary, our research results indicated that ZJC inhibited the growth of tumor.

The results of H&E staining showed that in the control group, the cells in the tumor tissue were arranged closely and disorderly, with large and deeply stained nuclei and obvious atypia. In the ZJC-L, ZJC-H and anti-PD-1 group, the tumor tissue cells were arranged loosely, and the nuclear atypia was significantly decreased. The immunohistochemical staining results of Ki67 also showed that compared with the control group, the proportion of Ki67-positive cells in the ZJC-L group, ZJC-H group, and anti-PD-1 group was significantly lower than that in the control group. This showed that the proliferative activity of tumor cells was reduced, further demonstrating the inhibitory effects of ZJC and anti-PD-1 on tumor growth (**Figure 6I**).

### ZJC exerts antitumor effects through the mTOR signaling pathway

**Figure 7:**
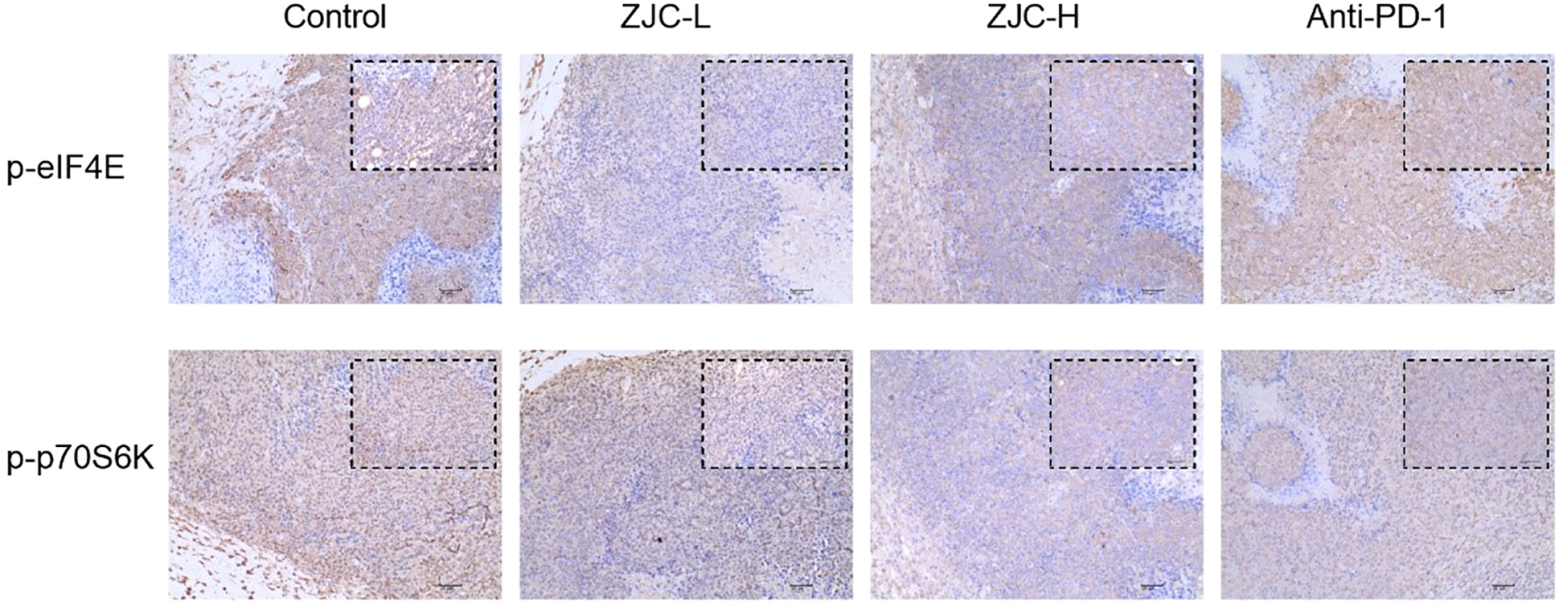
ZJC exerts anti-tumor effects through the mTOR signaling pathway Immunohistochemical staining images of p-eIF4E and p-p70S6K proteins in different groups.

These results showed that there were differences in the expression of p-eIF4E and p-p70S6K in tumor tissues of different groups. In the control group, the activity of p-eIF4E in tumor cells was relatively high. In contrast, in the ZJC-L, ZJC-H, and anti-PD-1 groups, the intensity of p-eIF4E positive staining was weakened, and the number of positive cells decreased. In the control group, p-p70S6K also showed strong positive staining, and a large number of brown positive signals could be seen in the tumor cells. In the ZJC-L, ZJC-H and anti-PD-1 groups, the positive staining intensity of p-p70S6K was weakened, the number of positive cells decreased, and the expression of p-p70S6K was inhibited (**Figure 7**).

### Immune infiltration analysis

**Figure 8:**
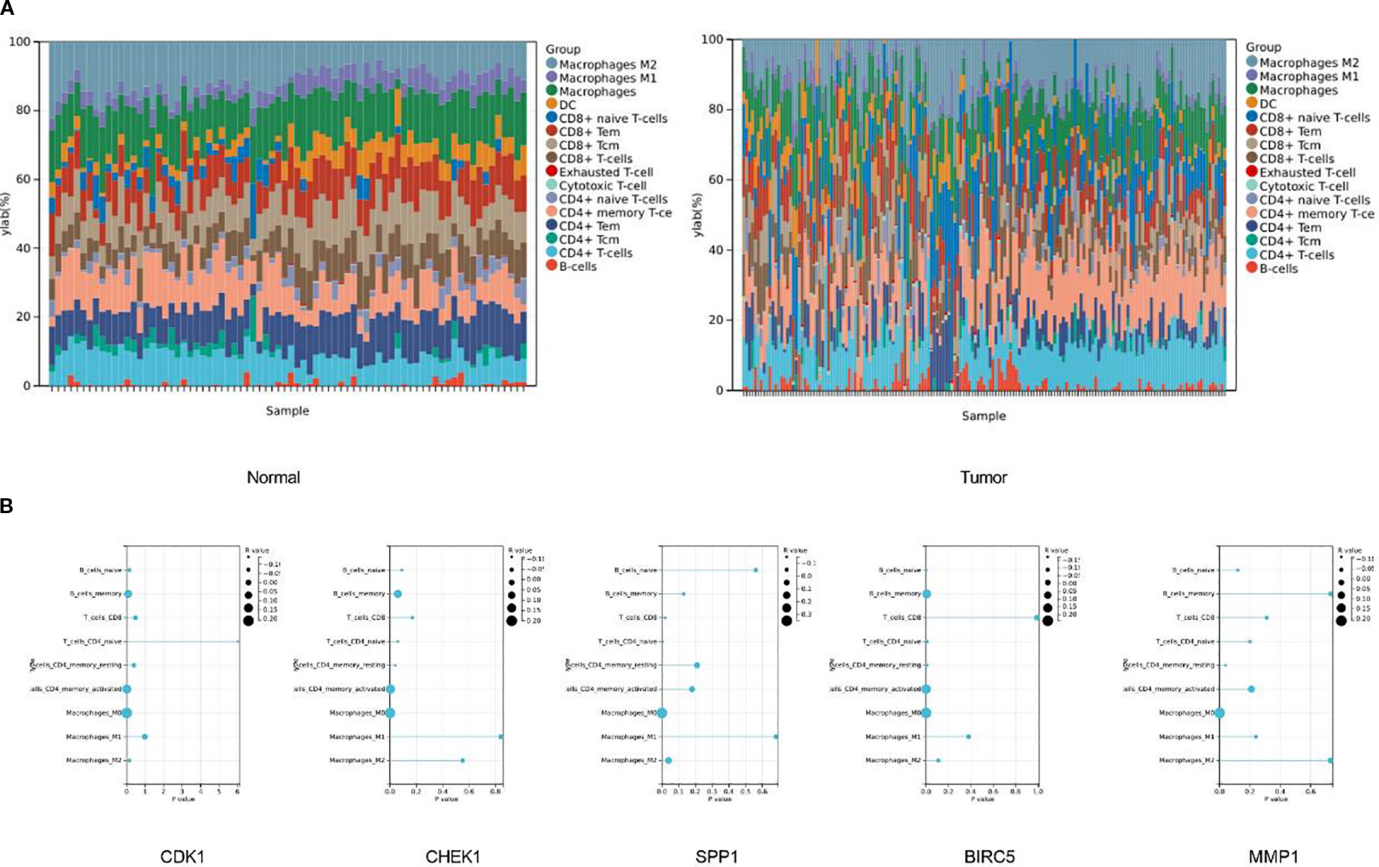
There are significant differences in the infiltration of immune cells between tumor tissues and normal tissues. **(A)** The stacked bar chart shows the proportions of different immune cell types in normal tissues (left chart) and tumor tissues (right chart). The vertical axis represents the percentage of cell proportions, and the horizontal axis represents the samples. **(B)** The correlations between five key genes and different immune cells in liver cancer.

In addition, to further clarify the characteristics of immune cell infiltration in normal liver tissues and HCC tissues, we evaluated the infiltration ratios of immune-related cells and the correlations among immune cells. The results of the stacked bar chart indicated that compared with normal tissues, in HCC tissues, the proportion of M1 macrophages decreased, while the proportions of M2 macrophages and exhausted T-cells increased (**Figure 8A**).

To further clarify the correlation between the target genes and immune cells, we conducted a correlation analysis between the top five key genes and immune cells. The results of the immune infiltration analysis indicated that CDK1 had a significant negative correlation with naïve CD4^+^ T cells (*P* = 6.0×10^-3^, r = -0.14), and significant positive correlations with activated memory CD4^+^ T cells (*P* = 5.7×10^-3^, r = 0.14) and M0 macrophages (*P* = 5.9×10^-5^, r = 0.21). CHEK1 showed a significant negative correlation with resting memory CD4^+^ T cells (*P* = 4.0×10^-2^, r = -0.11), and significant positive correlations with activated memory CD4^+^ T cells (*P* = 3.5×10^-3^, r = 0.15) and M0 macrophages (*P* = 1.3×10^-4^, r = 0.20). SPP1 had significant negative correlations with CD8^+^ T cells (*P* = 2.0×10^-2^, r = -0.13) and naïve CD4^+^ T cells (*P* = 5.0×10^-3^, r = -0.15), and significant positive correlations with M0 macrophages (*P* = 4.4×10^-12^, r = 0.35) and M2 macrophages (*P* = 4.0×10^-2^, r = 0.11). BIRC5 had significant negative correlations with naïve B cells (*P* = 1.2×10^-3^, r = -0.17), naïve CD4^+^ T cells (*P* = 1.0×10^-2^, r = -0.13), and resting memory CD4^+^ T cells (*P* = 8.6×10^-3^, r = -0.14), and significant positive correlations with memory B cells (*P* = 5.4×10^-3^, r = 0.15), activated memory CD4^+^ T cells (*P* = 4.2×10^-4^, r = 0.18), and M0 macrophages (*P* = 5.9×10^-6^, r = 0.24). MMP1 had a significant negative correlation with resting memory CD4^+^ T cells (*P* = 4.0×10^-2^, r = -0.11) and a significant positive correlation with M0 macrophages (*P* = 4.9×10^-5^, r = 0.21) (**Figure 8B**).

### ZJC improves the tumor immune microenvironment

**Figure 9:**
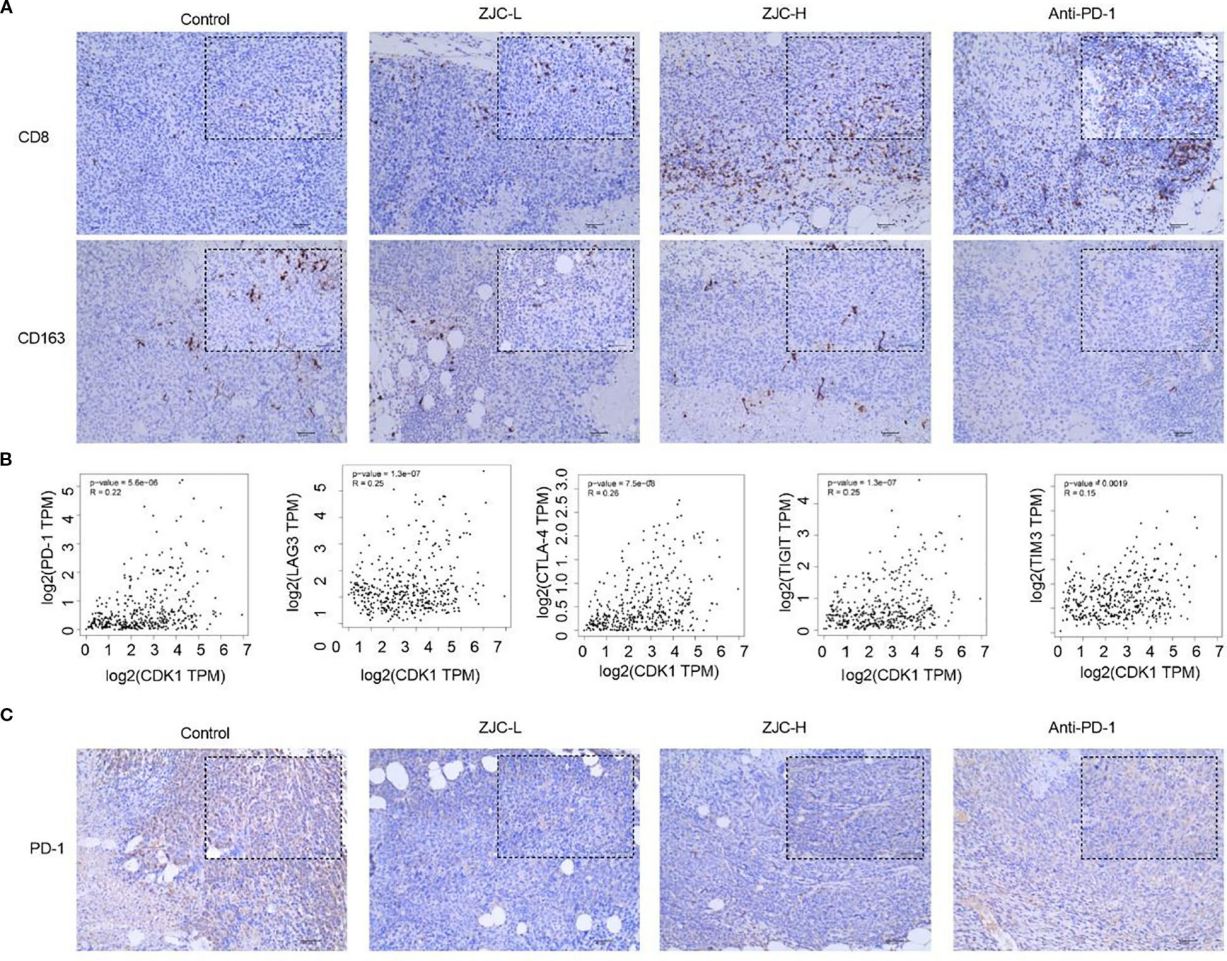
ZJC promotes the infiltration of CD8 T cells while reducing the abundance and CD163 macrophages. **(A)** The expression levels of CD8 and CD163 cells in different treatment groups. **(B)** The correlation between CDK1 and PD-1, CDK1 and LAG-3, CDK1 and CTLA-4, CDK1 and TIGIT, CDK1 and TIM-3. **(C)** The expression levels of PD-1 in different treatment groups.

The results showed that there were significant differences in the number of CD8 and CD163 among different treatment groups (control, ZJC-L, ZJC-H, anti-PD-1). There were fewer CD8 positive cells in the control group, while there were more positive cells in the ZJC-L, ZJC-H and anti-PD-1 groups. This indicated that ZJC and anti-PD-1 promoted the expression of CD8 cells (**Figure 9A**).

Previous studies have shown that high levels of M2-specific CD163 are associated with an increase in tumor nodules and poor prognosis in HCC patients (44). In this study, CD163 was used to label M2-type macrophages. The results showed that the positive cells of CD163 in the control group were relatively higher. Compared with the control group, there were fewer positive staining in the ZJC-L, ZJC-H and anti-PD-1 groups. This indicated that ZJC and anti-PD-1 inhibited the expression or aggregation of CD163-positive cells (**Figure 9A**).

Exhausted T cells often express high levels of inhibitory receptors, including PD-1, CTLA-4, TIGIT, TIM-3, LAG-3 (45–47). They may also express multiple inhibitory receptors simultaneously and suppress the T-cell immune response to tumor antigens (45–47). Therefore, we further analyzed the relationship between CDK1 and these inhibitory receptors. The GEPIA 2 database showed that there is a positive correlation between CDK1 and PD-1 (*P* = 5.6×10^-6^, r = 0.22), CDK1 and LAG-3 (*P* = 1.3×10^-7^, r = 0.25), CDK1 and CTLA-4 (*P* = 7.5×10^-8^, r = 0.26), CDK1 and TIGIT (*P* = 1.3×10^-7^, r = 0.25), CDK1 and TIM-3 (*P* = 1.9×10^-3^, r = 0.15) (**Figure 9B**).

The results of immunohistochemical staining showed that in the tumor tissues of the control group, the number of PD-1 positive staining was relatively large. Compared with the control group, the positive expression of PD-1 in the ZJC-L, ZJC-H and anti-PD-1 group also showed a downward trend in PD-1 expression (**Figure 9C**). These results showed that ZJC improves the tumor immune microenvironment in HCC.

## Discussion

**Figure 10:**
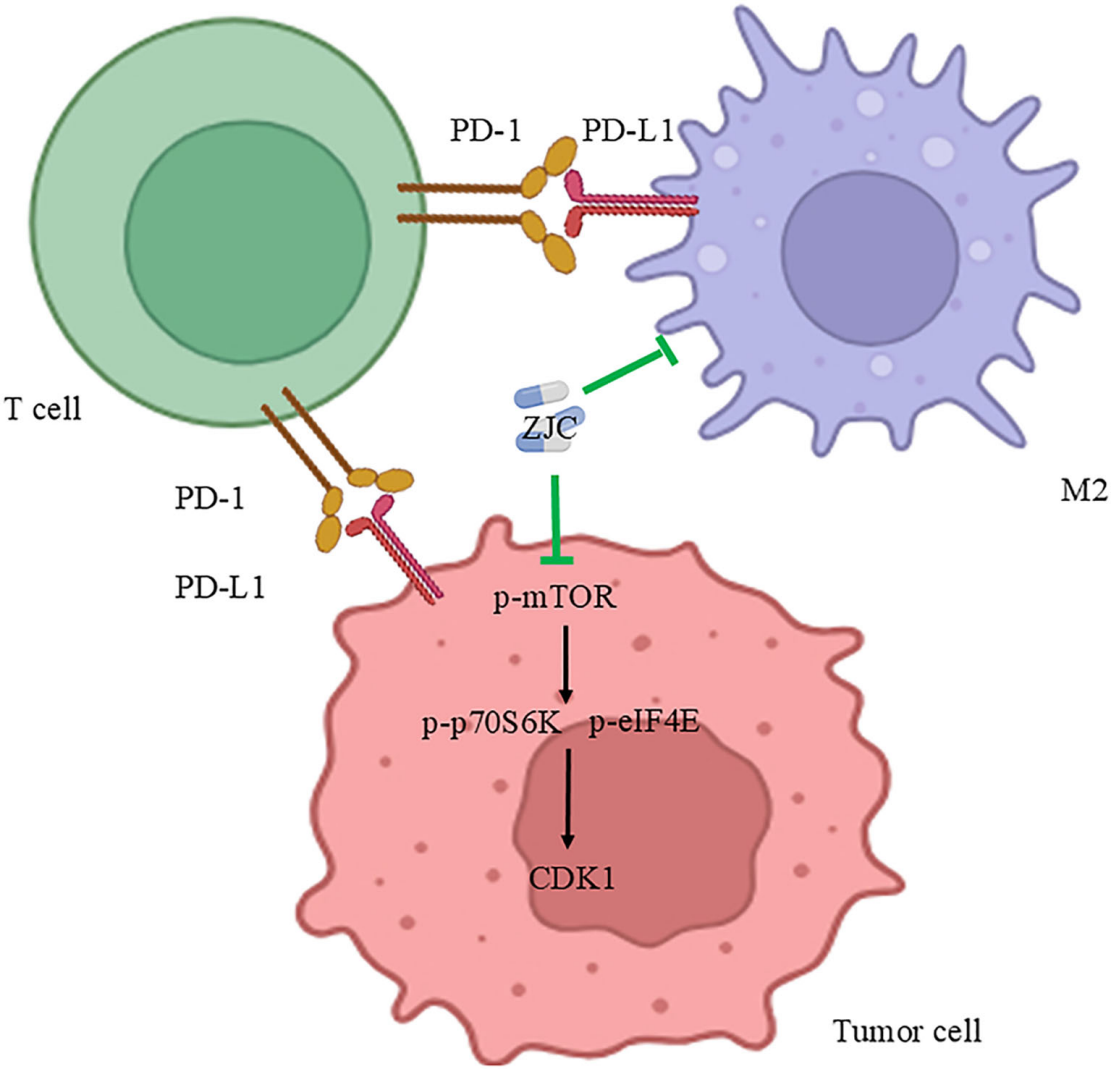
Mechanism diagram of ZJC’s anti-tumor effect. This schematic diagram illustrates the mechanism of the anti-tumor effect of ZJC. It demonstrates the interactions among T cells, tumor cells and M2-type macrophages through the PD-1/PD-L1 pathway. ZJC inhibits the mTOR pathway, including suppressing the expression of p-mTOR, thereby down-regulating the expressions of p-p70S6K and p-eIF4E.

This study systematically revealed the multi-dimensional mechanism by which ZJC improves the tumor immune microenvironment and inhibits HCC proliferation by targeting the mTOR signaling pathway and regulating CDK1 (**Figure 10**). This discovery provides evidence for the molecular mechanism of the anti-tumor effect of traditional Chinese medicine.

HCC is a common malignant tumor worldwide, with high incidence and mortality rates, posing a serious threat to human health. It is of great significance to find effective treatment strategies for liver cancer. This study obtained a large number of HCC targets by integrating multi-gene expression profile microarray data, laying a solid foundation for subsequent research. Tex plays a crucial role in tumor immune escape. Tex plays a crucial role in tumor immune escape. In this study, Tex targets were obtained and integrated from the GeneCards database and OMIM database, which is of great significance for analyzing the tumor immune microenvironment and the immunomodulatory effects of ZJC.

Based on the traditional Chinese medicine database and relevant screening criteria, the active ingredients and their targets in *Coptidis Rhizoma* and *Evodiae Fructus* were determined, and finally, the targets of ZJC were obtained. By taking the intersection, the key targets related to the treatment of HCC by ZJC and Tex were identified. *In vitro* cell experiments strongly confirmed the significant inhibitory effect of ZJC on the proliferation of Hep3B and HepG2.2.15 cells and determined its IC50. This result is similar to the inhibitory effect of ZJC on cancer cells in previous studies. Studies have shown that Zuojin pills (ZJP) significantly inhibit the G0/G1 phase and inhibit cell migration and invasion in a dose-dependent manner (15). ZJP also inhibited the proliferation of pancreatic cancer cells, inhibited the transition from G1 phase to S phase, and induced cell apoptosis (48). Notably, as observed in **Figure 2**, ZJC appears to promote the proliferation of HepG2.2.15 cells at lower concentrations (25-100µg/mL). It is speculated that this may be attributed to the following reasons: traditional Chinese medicine compound prescriptions have bidirectional regulatory effects (49, 50). As a compound, the biological effects of ZJC are concentration-dependent and bidirectional. At low concentrations, some components may weakly activate cell metabolic pathways and temporarily promote proliferation. At high concentrations, the cytotoxicity of the main components, such as inhibiting DNA synthesis and inducing apoptosis, is dominant, manifested as proliferation inhibition. HepG2.2.15 cells, due to the presence of integrated HBV genomes and viral antigens, may be more sensitive to certain trace components in ZJC. At low concentrations, these components may briefly activate proliferation-related pathways such as PI3K/Akt. At high concentrations, the overall inhibitory effect of ZJC, such as the down-regulation of the mTOR pathway, covers this activation. In addition, low-concentration ZJC may generate weakly irritating intermediate products through cellular metabolism or induce short-term adaptive proliferation of cells, while high-concentration drugs exceed the compensatory capacity of cells and eventually show inhibition. Further verification can be achieved by detecting the expression of proliferation-related proteins at low concentrations in the future.

In the aspect of mechanism research, GO and KEGG analyses revealed that these intersection targets were significantly enriched in multiple biological processes, cellular components, and molecular functions, and played a role in several important signaling pathways. Among them, the mTOR signaling pathway holds a central position in tumorigenesis and tumor development. It is involved in regulating processes such as cell growth, proliferation, and metabolism. In this study, in both Hep3B and HepG2.2.15 cells, ZJC could significantly reduce the expression of p-mTOR and its downstream key molecules p-eIF4E and p-p70S6K. Its effect was similar to that of the mTOR inhibitor INK128, and the mTOR activator MHY1485 could reverse this inhibitory effect. This fully demonstrates that ZJC exerts its anti-tumor effect by inhibiting the mTOR signaling pathway. This is consistent with the reported mechanisms of action of mTOR inhibitors in cancer treatment in other studies. For example, rapamycin and INK128, as mTOR inhibitors, have been widely used in pre-clinical and clinical studies of tumors (51, 52). Research has shown that the mTOR signaling pathway is over-activated in various tumors, and the inhibition of the mTOR pathway by INK128 can reverse the progression and metastasis of HCC (53).

Furthermore, we further identified five key targets, including CDK1, as important targets for ZJC to inhibit HCC. These targets were highly expressed in liver cancer tissues and were closely related to tumor staging and patient survival. *In vitro* experiments indicated that ZJC could inhibit the expression of CDK1. Studies have shown that CDK1, as a key protein in cell-cycle regulation, plays an important role in the proliferation of tumor cells. Inhibiting the activity of CDK1 induced cell-cycle arrest and apoptosis in tumor cells (32, 54). CDK1 was frequently enhanced in up to 46% of HCC tissues, which was significantly associated with poor OS. The CDK1 inhibitor RO3306 blocked the CDK1/PDK1/β-Catenin signaling to improve the efficacy of sorafenib treatment (55). In addition, the other four key genes also play important roles in promoting the occurrence and development of HCC. CHEK1 is a serine/threonine protein kinase that plays a crucial role in the cell’s response to DNA damage. When the integrity of the genome is compromised, the function of CHEK1 is to prevent DNA replication, thereby maintaining genomic stability and preventing abnormal cell division (56). This function is essential for inhibiting tumor formation. CHEK1 was overexpressed in HCC and was associated with poor OS. Inhibiting CHEK1 could weaken the invasion behavior and proliferation of HCC cells (57). SPP1 (also known as Osteopontin) is a bone sialoprotein involved in the attachment of osteoclasts to the mineralized bone matrix and is also a bone matrix protein. It is a multifunctional protein that can act on various receptors related to different signaling pathways associated with cancer (58). SPP1 promoted the proliferation and migration of HCC cells by increasing the production of reactive oxygen species (ROS) (59). BIRC5 was highly expressed in the vast majority of human cancers and was associated with chemoresistance, increased tumor recurrence, and shortened patient survival (60). Studies have shown that BIRC5 expression is highly correlated with the T-stage, pathological stage, histological grade, and AFP in HCC patients (61). BIRC5 silencing alleviated HCC by blocking the PPARγpathway and regulating cuprotosis, which may have therapeutic significance for HCC (62). The matrix metalloproteinases (MMPs), also known as matrix proteins, are a family of calcium-dependent endopeptidases that play key roles in cell recruitment, migration, differentiation, angiogenesis, and cell death (63). MMP1 is significantly elevated in HCC and is the main biomarker for the poor prognosis of HCC patients (64). These results showed that these five key genes played a vital role in HCC.

*In vivo* research results showed that ZJC could significantly inhibit the growth of liver cancer with relatively low toxicity. Histological and immunohistochemical analyses further confirmed this finding. ZJC inhibited the proliferative activity of tumor tissues and the expression of proteins related to the mTOR signaling pathway. This not only confirmed the anti-tumor effect of ZJC *in vivo*, but also verified the results of *in vitro* experiments. Similar studies have also shown that ZJC has a significant anti-tumor effect. Studies have found that ZJC inhibits the growth of colorectal cancer cells and tumors (9). In addition, ZJP regulated precancerous lesions of gastric cancer by inhibiting the mitogen-activated protein kinase kinase (MEK)/extracellular signal-regulated kinase (ERK)/MYC proto-oncogene, BHLH transcription factor (C-MYC) pathway and regulating cell proliferation and apoptosis (65). ZJP also inhibited the growth of gastric cancer cells by activating the mitochondrial-dependent apoptosis pathway (66).

Immune infiltration analysis revealed significant differences in immune cell infiltration between normal liver tissues and HCC tissues. In HCC tissues, the number of M1-type macrophages decreases, while the number of M2-type macrophages and exhausted T cells increase, which is closely related to tumor immune escape and poor prognosis. The role of ZJC in improving the tumor immune microenvironment is also worthy of attention. In animal models, both ZJC and anti-PD-1 inhibited tumor growth. However, its regulatory effects on exhausted T cells and M2 macrophages suggest that the mechanism by which it enhances anti-tumor immunity may be different from the PD-1/PD-L1 axis, providing experimental evidence for the combined use of traditional Chinese medicine and immune checkpoint inhibitors. By analyzing the correlation between key genes and immune cells, it was found that the target genes of ZJC were closely associated with various immune cells, indicating that ZJC may play an anti-tumor role by regulating the function and infiltration of immune cells and remodeling the tumor immune microenvironment. A study has shown that ZuoJin Wan (ZJW), a traditional pill, regulates a series of cells in the TME, including malignant colorectal cancer cells, immune cells, and stromal cells, while ZJC is a modern capsule with identical components and efficacy but different formulation. In colorectal cancer cell lines, the down-regulation of tissue inhibitor of matrix metalloproteinase 1 (TIMP1) and metadherin (MTDH) by ZJW might play an important role in the immunomodulation of colorectal cancer (67).

However, this study still has certain limitations. The relevant mechanism has not yet been verified in more clinical samples. Subsequent studies can further explore the efficacy and safety of ZJC in clinical applications. This study has not conducted in-depth exploration on the mechanism of the synergistic effect between ZJC and anti-PD-1. The synergistic effect has not been clarified through quantitative analyses such as combination index, and there is a lack of dose optimization research, making it impossible to determine the optimal combined medication scheme, which may affect the accurate evaluation of its clinical value. Future studies will establish a combination index model to clarify the mode of action, design multi-gradient dose experiments to screen the optimal ratio, and conduct in-depth exploration on the molecular mechanism of the synergistic effect. In addition, in this study, specific T cell subsets (such as CD4+, regulatory T cells) and their functional states have not been further validated using advanced techniques like flow cytometry and single-cell RNA sequencing, which results in an insufficient understanding of the fine regulatory mechanisms of T cells in the TME. Further verification will be carried out using advanced technologies such as flow cytometry and single-cell RNA sequencing. Moreover, the lack of tissue-specific detection and multi-time-point dynamic detection has rendered the interpretation of “tissue-system cytokine expression discrepancies” relatively preliminary, and this aspect will be strengthened in future studies.

In summary, this study systematically revealed the potential mechanism of ZJC in the treatment of HCC. Specifically, ZJC exerts its therapeutic effect by inhibiting the mTOR signaling pathway, regulating key targets such as CDK1, and thereby suppressing the proliferation of HCC cells. Meanwhile, ZJC also improves the tumor immune microenvironment, which further contributes to its anti-HCC efficacy (**Figure 10**). These findings provide new theoretical basis and potential treatment strategies for the treatment of HCC.

## Data Availability

All bioinformatics data used in this study were obtained from the Gene Expression Omnibus (GEO) and other online databases, with no new bioinformatics data generated in this research; meanwhile, the *in vivo* and *in vitro* experimental data can be obtained from the corresponding author upon reasonable request.

## Supplementary material

Supplementary Figure 1 PCA of transcriptomic datasets before and after batch effect correction.

Supplementary Figure 2 Molecular docking **(A)** .The molecular docking results of quercetin and CDK1. **(B)**.The molecular docking results of isorhamnetin and CDK1.

Supplementary Table 1 Molecular docking results of CDK1 and main active components (kcal/mol)
