# Supplementary figures and images for: Zuojin capsule improves T cell exhaustion and tumor immune microenvironment of hepatocellular carcinoma through the mTOR-eIF4E/p70S6K-CDK1 pathway
Source: Front Immunol. 2025 Sep 22;16:1617604. doi: 10.3389/fimmu.2025.1617604 (PMC12497796; doi:10.3389/fimmu.2025.1617604)

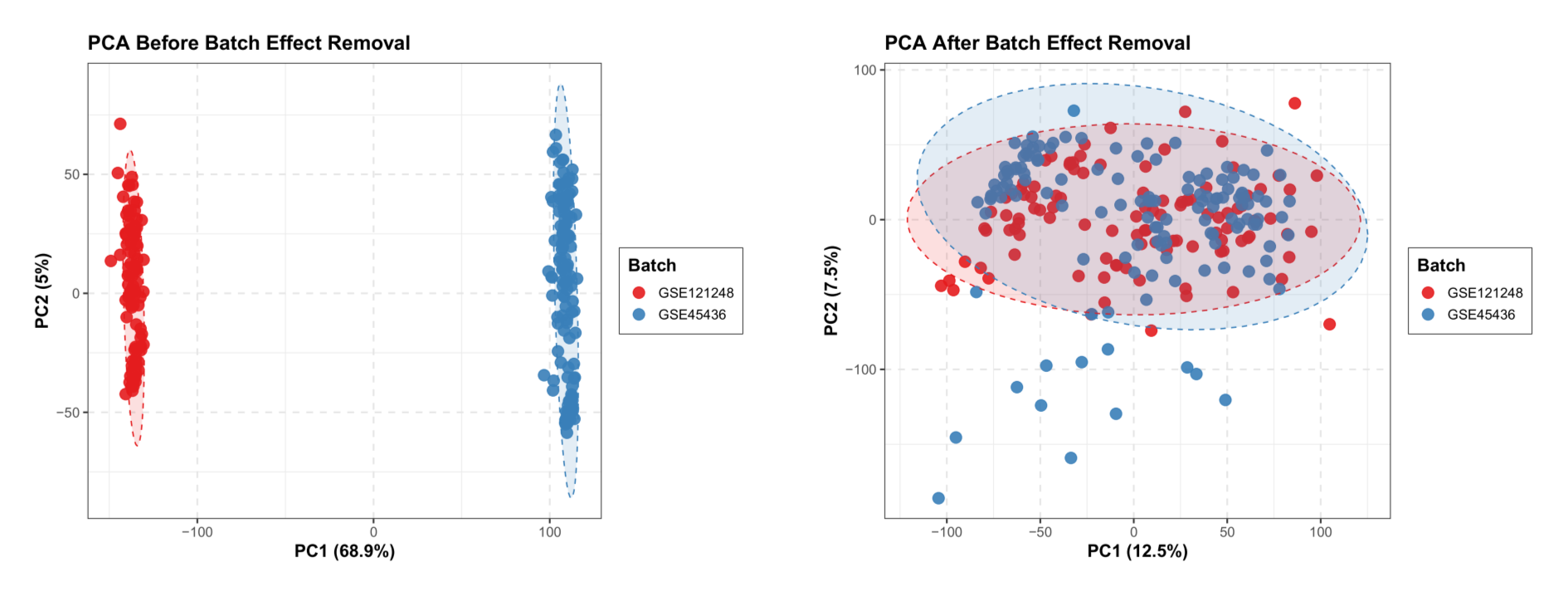

Supplement: Supplementary Figure 1 — PCA of transcriptomic datasets before and after batch effect correction. [file Image1.tif]

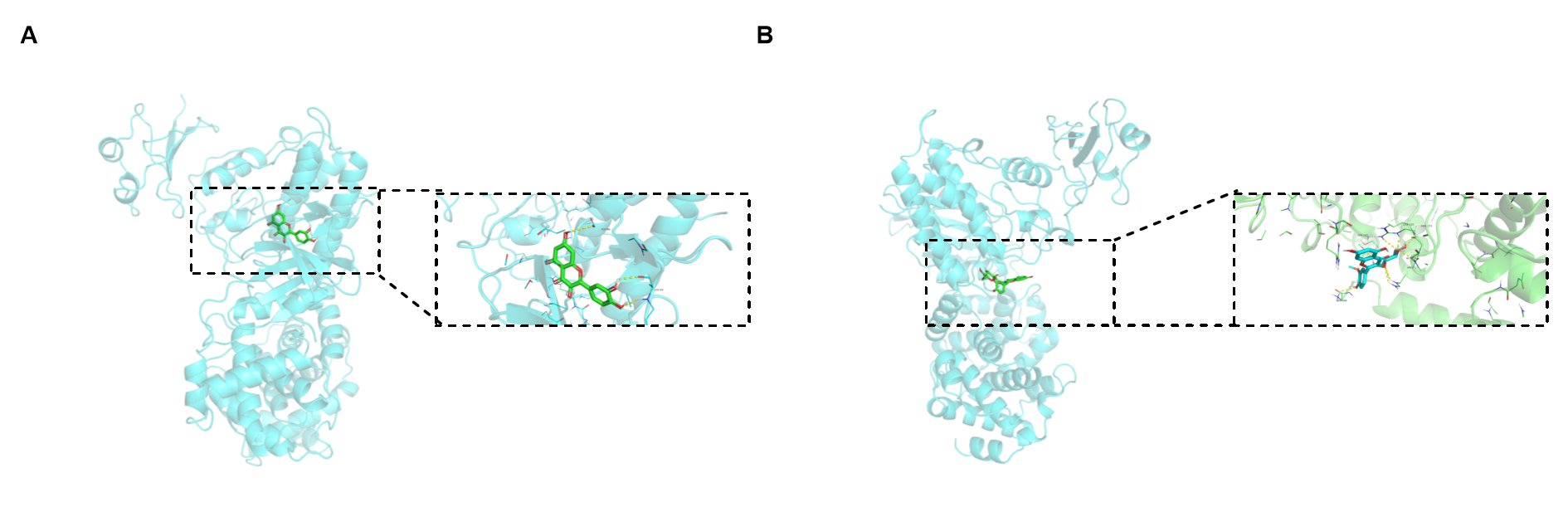

Supplement: Supplementary Figure 2 — Molecular docking (A) .The molecular docking results of quercetin and CDK1. (B).The molecular docking results of isorhamnetin and CDK1. [file Image2.tif]
