# Supplementary material for: Zuojin capsule improves T cell exhaustion and tumor immune microenvironment of hepatocellular carcinoma through the mTOR-eIF4E/p70S6K-CDK1 pathway
Source: Front Immunol. 2025 Sep 22;16:1617604. doi: 10.3389/fimmu.2025.1617604 (PMC12497796; doi:10.3389/fimmu.2025.1617604)
Supplement: Supplementary Table 1 — Molecular docking results of CDK1 and main active components (kcal/mol) [file Table1.docx]

**Supplementary Table 1.** **Molecular docking results of CDK1 and main active components (kcal/mol)**

| Ligands/Receptor | Binding energy |
| --- | --- |
| quercetin | -9.4 |
| isorhamnetin | -8 |
| berberine | -6.2 |
| betasitosterol | -5 |
